# Is there a weekend effect in severe traumatic brain Injury? An observational, population-based study from the Norwegian Trauma registry

**DOI:** 10.1016/j.bas.2025.104263

**Published:** 2025-04-28

**Authors:** Joakim Stray Andreassen, Mattis A. Madsbu, David Andreas Thomas Werner, Clemens Weber

**Affiliations:** a Department of Neurosurgery, St. Olavs University Hospital, Trondheim, Norway; b Department of Circulation and Medical Imaging, Faculty of Medicine and Health Sciences, Norwegian University of Science and Technology (NTNU), Norway; c Department of Neurosurgery, Stavanger University Hospital, Stavanger, Norway

**Keywords:** Epidemiology, Traumatic brain injury, Mortality, Weekend

## Abstract

**Introduction:**

Traumatic brain injury is prevalent and with severe injury the mortality is high. The aim of this study was to explore differences in mortality rates between weekday and weekend admissions for patients with severe traumatic brain injury (TBI) in Norway based on data from the National Trauma Registry of Norway (NTR).

**Research question:**

Are mortality rates higher for patients with severe traumatic brain injury admitted on weekends compared to weekdays?

**Materials and methods:**

This study is an observational cohort study based on prospectively collected, population-based data from the NTR. The study period was between January 01, 2017 and December 31, 2020 and included all patients with severe TBI registered in the database.

**Results:**

During the 4 years-study period 627 patients with severe TBI were identified; the median age was 53.0 years (IQR 32.0-71.0). Weekend admissions involved significantly younger patients with a median age of 50.0 years compared to a median age of 57.0 years on weekdays (p = 0.013). Overall, the 30-day mortality rate was 39 %, with higher mortality observed on weekdays compared to weekends (43 % vs. 34 %; p = 0.025). Logistic regression analysis showed that age and higher AIS head injury severity were significantly associated with 30-day mortality, while admission timing (weekend vs. weekday) showed no association.

**Discussion and conclusion:**

Weekend admissions in severe TBI are frequent and involves younger patients compared to weekdays. Our study did not find a weekend effect on the mortality rate for severe TBI in Norway based on data from the NTR.

## Highlights

• Severe traumatic brain injuries has high mortality and affects young patients.
• Severe traumatic brain injuries occur frequently on weekends.
• Mortality rates are not affected by admissions on weekends compared to weekdays.
• “Weekend-effect” was not identified in this study.

## 1 Introduction

Traumatic brain injury (TBI) has the highest incidence among common neurological disorders and is a leading cause of injury-related deaths worldwide (Maas et al., 2022). TBI leads to approximately 2 million hospital admissions and 82,000 deaths each year in Europe (Maas et al., 2022; Majdan et al., 2016). The incidence rate of TBI is challenging to interpret due to variations in inclusion criteria and case definitions. In Europe an incidence rate of 262 per 100,000 was reported in 2016, however a current living systematic review shows a vast range in numbers from country to country ranging from 83.3 to 849 per 100.000 highlighting the difficulty of reporting incidence rates (Peeters et al., 2015; Brazinova et al., 2015). TBI mortality depends on several factors such as severity of injury, age and health of patients and quality of medical care given. Mortality from severe TBI remains high and with substantial differences across studies it reaches up to 46 % (Walder et al., 2013; Andriessen et al., 2011; Andreassen et al., 2022).

Admission of patients with severe TBI reaches a peak during weekends, typically affecting younger individuals. Intoxication with drugs or alcohol and road traffic incidents (RTI) are frequently observed in these cases (Andreassen et al., 2022; Bjarkø et al., 2019). The term “weekend effect” describes increased morbidity and mortality related to a given disorder observed during the period Saturday to Sunday. “Weekend effect” has been related to various disorders such as stroke, acute epiglottitis, pulmonary embolism, ruptured aortic aneurysm, and cardiovascular emergencies (Bell and Redelmeier, 2001; Kostis et al., 2007; Mekonnen et al., 2020; Kim et al., 2023). Studies on trauma patients admitted during weekends have yielded mixed results, showing both increased and decreased mortality rates (Andreassen et al., 2022; Schneider et al., 2012; Posti et al., 2021; Ko et al., 2023; Carr et al., 2011). However, there is limited evidence regarding the weekend effect in TBI. A Finnish study found increased TBI-related hospital admissions and 30-day mortality after TBI on public holidays and weekends (Posti et al., 2021). Another study from the US reports higher mortality in older adults with TBI admitted on weekends (Schneider et al., 2012). We previously investigated a possible weekend effect in TBI based on data from a regional trauma database, but no differences were detected with regards to mortality (Andreassen et al., 2022).

The aim of this study was to investigate a potential weekend effect on the mortality of severe TBI in Norway based on nationwide data from a national trauma registry.

## 2 Methods

### 2.1 Study design and period

This study is an observational cohort study based on prospectively collected, population-based data from the National Trauma Registry of Norway (NTR). The study period was between January 01, 2017 and December 31, 2020 and included all patients with severe TBI registered in the database.

### 2.2 Ethics

The study followed the STROBE guidelines for observational research (von Elm et al., 2008). Ethics approval was granted by The Regional Committee for Medical and 10.13039/100005622Health Research Ethics of 10.13039/100007159Western Norway (REK143902/2020). The study was approved by the Scientific Council and the patient representative of the NTR.

### 2.3 Data collection

The NTR is a mandatory trauma registry covering the entire population of Norway, which has a public, single-payer health-care system with no private hospitals delivering trauma care. Public acute care hospitals in Norway are divided into two groups: regional trauma centers and hospitals with acute care trauma function (Choi et al., 2022; Bredin et al., 2022). The registry gathers data from all 38 public hospitals admitting trauma patients, four hospitals are regional trauma centers and 34 are hospitals with acute care trauma function (Bredin et al., 2022). In Norway there are 5 hospitals delivering acute neurosurgical trauma care and treating patients with severe TBI. Norway has a population of 5.4 million people, a population density of 15/km^2^, an urban population of 83 %, and a life expectancy of 82.9 years (as per December 2020) (S, 2020).

Data completeness in the NTR is generally high for different variables demonstrating 98.7 % for patient age, 99.7 % for type, mechanism and intention of injury, 100 % for sex and 99.7 % for outcome variables. There is also excellent agreement with data from NTR compared to corresponding electronic patient records (Naberezhneva et al., 2023). The data presented in this study represents nearly complete, population-based data for the whole country.

### 2.4 Study population

This study includes all patients with severe TBI admitted to hospitals in Norway over a 4-year period. TBI is often categorized into minimal, mild, moderate, and severe according to the Head Injury Severity Scale (HISS), where severe TBI is defined as Glasgow Coma Scale (GCS) score between 3 and 8 (Stein and Spettell, 1995). Using GCS 3–8 as a stand-alone marker for severe TBI has several limitations as an altered GCS may be influenced by pre-hospital procedures, medications, alcohol or drug intoxication, injuries to the face, stroke and various medical conditions. Another score for assessing brain injury is the Abbreviated Injury Scale (AIS) score for head injuries, which ranges from 1 to 6, with higher number indicating greater severity (Palmer et al., 2016). The literature offers multiple definitions of severe TBI and the main parameters are GCS and/or AIS, but there is no agreement on optimal cut-off for AIS score with to define severe TBI (Savitsky et al., 2016). To include only patients with severe TBI from the database and to exclude patients with a GCS score <9 due to other causes, the patients for this study were identified in the database by the following variables: GCS score between 3 and 8, AIS score for head injuries of 3 or more and Injury Severity Score (ISS) ≥ 13 (Palmer et al., 2016; Teasdale and Jennett, 1974).

### 2.5 Variables and outcome

Patient demographics, data and injury characteristics were recorded including type of injury (blunt versus penetrating) and mechanism of injury (RTI, hit by blunt object, low energy fall, high energy fall, other). Injury severity (GCS, ISS, AIS, etc.) and treatment variables such as length of hospital-stay and ventilator treatment were recorded. Outcome was defined as 30-day mortality after injury and discharge destination after acute care (home, rehabilitation, other ICU, another hospital ward, other).

### 2.6 Injury dates

The injury dates were registered according to the day of the week the injury occurred and were dichotomized into weekdays (Monday-Friday) and weekends (Saturday-Sunday).

### 2.7 Statistical analysis

All statistical analyses were performed with SPSS version 26.0 (IBM, USA). The chi square test was used for categorical variables and independent samples T-test was used for continuous variables. To describe the patient cohort medians, percentages and interquartile range (IQR) were utilized. Logistic regression analysis was performed to examine the association of different variables on the 30-day mortality rate of severe TBI. The regression model included four different variables (age, gender, weekday admission, AIS head injury severity). Statistical significance was defined as p ≤ 0.05.

## 3 Results

Over the 4-year study period, a total of 627 patients with severe TBI were identified from the NTR. The median age of the cohort was 53.0 years (IQR 32.0-71.0), with 74 % of patients being male. More patients were admitted on weekdays (363 patients, 58 %) compared to weekends (264 patients, 42 %). Patients admitted during weekends were significantly younger (50.0 years (IQR 29.5-65.0); p = 0.013) compared to those admitted on weekdays (57.0 years (IQR 33.0-74.0)), hence weekday admissions demonstrated significantly higher proportion of patients over 65 years (p < 0.001). Patient characteristics are presented in Table 1.

**Table 1:** Patient and injury characteristics of severe traumatic brain injury.

Variables	All patients	Weekday	Weekend	p-value
No. of patients	627	363	264	
Age in years, median (IQR)	53,0 (32,0–71,0)	57,0 (33,0–74,0)	50,0 (29,5–65,0)	*0,013*
Age over 65 years, No (%)	212 (34 %)	143 (40 %)	69 (27 %)	*<0,001*
Gender, No. (%)				
Male	463 (74 %)	264 (73 %)	199 (75 %)	0,456
Female	164 (26 %)	99 (27 %)	65 (25 %)	
Type of injury, No. (%)				
Blunt	614 (98 %)	355 (98 %)	259 (99 %)	0,475
Penetrating	11 (2 %)	7 (2 %)	4 (1 %)	
Mechanism of injury, No. (%)				
RTA	198 (32 %)	114 (32 %)	84 (32 %)	0,920
Hit by blunt object	47 (8 %)	27 (8 %)	20 (8 %)	1000
Low energy fall	148 (24 %)	92 (25 %)	56 (21 %)	0,231
High energy fall	201 (32 %)	106 (29 %)	95 (36 %)	0,071
Other	31 (5 %)	23 (6 %)	8 (3 %)	0,064

IQR – Interquartile range; RTA – Road traffic accident.

The primary mechanisms of injury were falls (both low and high energy), encompassing more than half of all injuries (56 %), followed by RTI (32 %). Injury severity was comparable between patients admitted on weekdays and weekend (see Table 2).

**Table 2:** Injury severity and length of treatment.

Variables	All patients	Weekday	Weekend	p-value
No. of patients				
GCS, median (IQR)	6 (3–9)	6 (3–9)	6 (3–10)	0,961
Systolic blood pressure, median (IQR)	134 (112–152)	135 (115–156)	132 (110–150)	0,882
ISS, median (IQR)	26 (25–34)	26 (25–35)	26 (21–33)	0,227
NISS, median (IQR)	42 (27–54)	41 (27–54)	43 (29–56)	0,730
AIS head injury severity, No. (%)				
AIS 3 (serious)	108 (17 %)	65 (18 %)	43 (16 %)	0,565
AIS 4 (severe)	135 (22 %)	71 (20 %)	64 (24 %)	0,175
AIS 5 (critical)	377 (60 %)	222 (61 %)	155 (59 %)	0,454
AIS 6 (maximal)	4 (1 %)	2 (1 %)	2 (1 %)	0,565
LoS, median (IQR)	3 (1–10)	4 (1–9)	3 (1–11)	0,620
LoVT, median (IQR)	3 (1–11)	3 (2–9)	3 (1–13)	0,452

ISS – Injury Severity Score; IQR – Interquartile range; NISS – New Injury Severity Score; AIS – Abbreviated Injury Score; LoS – Length of hospital stay; LoVT – Length of ventilator treatment.

Outcome data are presented in Table 3. The 30-day mortality for the whole cohort was 39 % (n = 246). Weekday admissions demonstrated overall higher mortality (43 % versus 34 %, p = 0.025). The mortality rates for patients <65 years were 31 % on weekdays vs 23 % on weekends (p = 0.181), while for those aged >65 years it was 63 % on weekdays vs. 67 % on weekends (p = 0.784), as shown in Table 4.

**Table 3:** Outcome variables of severe traumatic brain injury.

Variables	All patients	Weekday	Weekend	p-value
30-day mortality, No. (%)	246 (39 %)	156 (43 %)	90 (34 %)	*0,025*
Discharge destination, No. (%)				
Home	62 (10 %)	32 (9 %)	30 (11 %)	0,289
Rehabilitation	132 (21 %)	74 (20 %)	58 (22 %)	0,699
Other ICU	130 (21 %)	70 (19 %)	60 (23 %)	0,289
Another hospital ward	45 (7 %)	23 (6 %)	22 (8 %)	0,338
Other	40 (6 %)	30 (8 %)	10 (4 %)	*0,024*

ICU – Intensive Care Unit.

**Table 4:** Outcome variables of severe TBI in patients over and under 65 years.

	Age <65			Age >65		
	**Weekday**	**Weekend**	**p-value**	**Weekday**	**Weekend**	**p-value**
No. of patients	216	190		142	69	
30-day mortality, No. (%)	66 (31 %)	44 (23 %)	0.181	90 (63 %)	46 (67 %)	0.784
Discharge destination, No. (%)						
Home	24 (11 %)	27 (14 %)	0.402	8 (6 %)	0	0.060
Rehabilitation	65 (30 %)	51 (27 %)	0.577	8 (6 %)	7 (10 %)	0.275
Other ICU	46 (12 %)	45 (23 %)	0.675	22 (15 %)	15 (22 %)	0.299
Another hospital ward	10 (5 %)	18 (10 %)	0.087	12 (9 %)	3 (4 %)	0.412
Other	8 (4 %)	7 (4 %)	1.000	21 (15 %)	3 (4 %)	0.047

Twenty-one percent of patients (n = 130) were discharged to a rehabilitation facility, as detailed in Table 3. Among patients under 65 years, a higher percentage were discharged directly to rehabilitation facilities compared to those aged 65 years and older, as presented in Table 4.

In the logistic regression analysis (Table 5) age and higher AIS head injury severity were associated with increased 30-day mortality whereas time of admission (weekend) showed no association.

**Table 5:** Binary logistic regression analysis for 30-day mortality.

Variables	p-value	OR	95 % CI for OR
30-day mortality			
Age	*<0,001*	0,97	0,96–0,98
Gender (male)	0,194	1,33	0,87–2,04
Weekday	0,196	0,78	0,54–1,14
AIS head injury severity	*<0,001*		
AIS 3 (serious)	0,25	0,67	0,34–1,33
AIS 4 (severe)	*<0,001*	0,2	0,11–0,36
AIS 5 (critical)	*0,002*	0,02	0,01–0,25

OR - Odds Ratio; CI - Confidence Interval.

## 4 Discussion

This nationwide trauma registry study revealed a mortality rate of 39 % in patients suffering severe TBI in Norway. Patients admitted on weekdays were older and had a higher 30-day mortality. However, when adjusting for different risk factors mortality did not differ between weekday and weekend admission, i.e. a weekend effect for severe TBI in Norway was not found.

Research findings on the weekend effect in patients with TBI present conflicting results. Posti et al. conducted a comprehensive study of close to 70.000 TBI patients (of all severities) in Finland, revealing a significantly higher mortality rate during weekend admissions (Posti et al., 2021). Patients admitted with acute traumatic subdural hematomas during weekends has been associated with a small, but statistical difference, however its clinical significance is considered to be uncertain due to some methodological limitations (Rumalla et al., 2017).

Schneider et al. analyzed TBI (AIS head of 3 and 4) among older adults (≥65 years) and weekend injury was associated with a 13 % increased risk of mortality (Schneider et al., 2012). Our study does not support this finding, but the fatal outcome in patients >65 years is in line with a large meta-analysis demonstrating a 65 % mortality rate in patients over 60 years with severe TBI (McIntyre et al., 2013).

Our research group previously conducted a study analyzing patients with moderate and severe TBI at a regional trauma center, revealing no significant difference in mortality between weekend and weekday admissions (Andreassen et al., 2022). These findings align with a recent meta-analysis, which similarly reported no significant differences (Jang and Jang, 2023). Studies on this subject often encompass TBI of all severities which may not necessitate emergency interventions compared to those with severe TBI. Thus, the urgency and timing of possible interventions likely has less impact on the majority of patients across these studies (Jang and Jang, 2023).

A weekend effect has been observed across different diagnoses for emergency admissions, with medical diagnoses predominating (Aylin et al., 2010). Additional studies into polytrauma patients have failed to demonstrate a clear correlation between weekend admissions and increased mortality, suggesting that the trauma system may mitigate any potential delays (Carr et al., 2010; Lin et al., 2023; Guly et al., 2006). A statewide study in the US including level I-III trauma centers, revealed lower mortality among patients presenting on weekends, with no delays observed for procedures such as craniotomy or laparotomy (Carr et al., 2011).

Studies reporting a weekend effect have raised questions regarding potential underlying causes, including whether injuries sustained during weekends tend to be more severe, whether resource limitations with reduced staffing and less access to specialized care is compromised (Schneider et al., 2012; Jung and Ryu, 2023; Little et al., 2019). In cases of severe TBI, patients are typically admitted via a trauma team activation. These admissions follow standardized protocols involving prehospital notification, check-list assessments, prompt access to imaging, involvement across multiple specialties and timely life-saving interventions. This organized framework is designed to reduces delays in both diagnosis and treatment, regardless of admission timing. Egol et al. investigated mortality at different levels of trauma and found overall higher mortality rate during nighttime (Egol et al., 2011). The association being weakest at level I and II and strongest at level III and IV centers. This may reflect the robustness of larger trauma centers in regards to staffing and availability of interventions at times of lower clinical activity (Egol et al., 2011). In Norway, patients with severe TBI are typically transported directly to regional trauma centers, which offer neurosurgical services and advanced resources not available at local hospitals. Consequently, patients with more severe injuries are admitted to hospitals less likely to exhibit weekend effect (Lin et al., 2023). Nationally, the proficiency of trauma team services is maintained through regular simulation training, conducted more frequently at regional trauma centers, and Advanced Trauma Life Support (ATLS) certification is a requirement for all trauma team leaders (Bredin et al., 2022; Søreide, 2008). The importance of regular trauma simulation training has proven effective to enhance trauma team function (Falcone et al., 2008).

Studies that demonstrate a weekend effect have questioned whether it can be injury related (more severe injuries occurring during weekends), resource availability with less staffing options during weekends, less availability of specialized care. Most studies suggest that well-organized trauma service can mitigate this.

## 5 Strength and limitations

This study is strengthened by the use of prospectively collected population-based data with high completeness and excellent agreement with patient records (Naberezhneva et al., 2023). The data presented encompasses the whole of Norway which increases the external validity of the study. Severe TBI solely defined by a GCS ≤8 may lead to false positives, particularly on weekends due to potential inflicting factors such as alcohol and drug intoxication. Incorporating additional scores (ISS and AIS) in the current study increases the likelihood of identifying “true” severe TBI. This likely enhances the generalizability for this patient population. In addition, all hospitals with trauma functions have been included.

However, there is no long-term data available for this patient cohort. Further, comparability with other studies is challenging due to variations of definitions used in severe TBI. Another limitation is the lack of data on surgical interventions and time from admission to surgery.

## 6 Conclusion

Our study did not find a weekend effect on the mortality rate for severe TBI in Norway based on data from the NTR.

## Ethics approval and consent to participate

Ethics approval was granted by The Regional Committee for Medical and 10.13039/100005622Health Research Ethics of 10.13039/100007159Western Norway (REK143902/2020). The study was approved by the Scientific Council and the patient representative of the NTR. All patients included in the NTR, and consequently included in this study, receive written information about the registry and the option to anonymize their data within the NTR.

## Funding

The authors received no funding for the study.

## Declaration of competing interest

The authors declare that they have no known competing financial interests or personal relationships that could have appeared to influence the work reported in this paper.
